# The Underlying Mechanism of *Paeonia lactiflora* Pall. in Parkinson’s Disease Based on a Network Pharmacology Approach

**DOI:** 10.3389/fphar.2020.581984

**Published:** 2020-11-23

**Authors:** Wanqing Du, Xiao Liang, Shanze Wang, Philip Lee, Yunling Zhang

**Affiliations:** ^1^ Graduate School, Beijing University of Chinese Medicine, Beijing, China; ^2^ Department of Neurology, Xiyuan Hospital, China Academy of Chinese Medical Sciences, Beijing, China; ^3^ Dongzhimen Hospital, Beijing University of Chinese Medicine, Beijing, China; ^4^ Dongfang Hospital, Beijing University of Chinese Medicine, Beijing, China

**Keywords:** Parkinson’s disease, Paeonia lactiflora Pall., network pharmacology, multi-target, Traditional Chinese Medicine, apoptosis

## Abstract

**Background:** Parkinson’s disease (PD) is the second most common neurodegenerative disease worldwide, yet as of currently, there is no disease-modifying therapy that could delay its progression. *Paeonia lactiflora* Pall. is the most frequently used herb in formulas for PD in Traditional Chinese Medicine and also a potential neuroprotective agent for neurodegenerative diseases, while its mechanisms remain poorly understood. In this study, we aim to explore the underlying mechanism of *P. lactiflora* in treating PD utilizing a network pharmacology approach.

**Methods:** The protein targets of *P. lactiflora* ingredients and PD were first obtained from several databases. To clarify the key targets, a Protein-Protein-Interaction (PPI) network was constructed and analyzed on the String database, and then enrichment analysis was performed by the Metascape platform to determine the main Gene Ontology biological processes and Kyoto Encyclopedia of Genes and Genomes pathways. Finally, the Ingredient-Target-Pathway (I-T-P) network was constructed and analyzed by Cytoscape software.

**Results:** Six active ingredients of *P. lactiflora* (kaempferol, ß-sitosterol, betulinic acid, palbinone, paeoniflorin and (+)-catechin) as well as six core targets strongly related to PD treatment [AKT1, interleukin-6, CAT, Tumor necrosis factor (TNF), CASP3, and PTGS2] were identified. The main pathways were shown to involve neuroactive ligand-receptor interaction, Calcium signaling pathway, PI3-Akt signaling pathway, TNF signaling pathway, and apoptosis signaling pathway. The main biological process included the regulation of neurotransmitter levels.

**Conclusion:** *P. lactiflora* may retard neurodegeneration by reducing neuroinflammation, inhibiting intrinsic and extrinsic apoptosis, and may improve motor and non-motor symptoms by regulating the levels of neurotransmitters. Our study has revealed the mechanism of *P. lactiflora* in the treatment of PD and may contribute to novel drug development for PD.

## Introduction

Parkinson’s disease (PD) is becoming the fastest growing neurological disorder surpassing Alzheimer’s disease (AD), with the number of patients expected to double to 14.2 million by 2040 (Dorsey and Bloem, 2018). The clinical features of PD are heterogeneous and complex, including typical motor symptoms such as bradykinesia, resting tremor, rigidity, and postural instability, as well as non-motor symptoms (NMS) such as hyposmia, sleep disorders, autonomic nervous dysfunction, and mental and cognitive disorders (Kalia and Lang, 2015). The objectives of treatment involve symptomatic treatment and disease-modifying therapy (de Bie et al., 2020). However, adverse events of symptomatic supplement with levodopa, such as dyskinesia, fluctuations, psychiatric symptoms are frequently observed in the advanced stage of the disease (National Institute for Health and Care Excellence, 2017), and drugs for NMS are quite limited (Seppi et al., 2019). At present, no disease-modifying therapy is available to delay the progression of the disease (Kalia and Lang, 2015), thus underlining the urgency to discover drugs to change the course of the disease and improve symptoms. In view of the complicated pathological mechanism of PD, including abnormal aggregation of α-synuclein, inflammation, oxidative stress, and apoptosis, novel drugs may need to target multiple pathways at the same time (Elkouzi et al., 2019).

For this reason, medicinal plants with multiple ingredients have received widespread attention for PD neuroprotection. A previous research has already summarized more than 50 types of plants against MPTP induced neurotoxicity (Abushouk et al., 2017), including *Mucuna pruriens* seeds and *P. lactiflora* roots. Many of these herbs were discovered in traditional medicine practice, and now verified by modern experiments to play a role in various pathological process of PD.

In China, where Traditional Chinese medicine (TCM) accounts for an incredible 28.55% of its total pharmaceutical industry (WHO, 2019), *P. lactiflora* is reported to be the most frequently used herb in the treatment of PD (Sun and Peng, 2017). The dried root of *Paeonia lactiflora* Pall., also known as *Paeoniae Radix Alba*, or Bai shao in Chinese, has been applied in TCM for 2000 years since first recorded in the Prescriptions for Fifty-two Aliments (Yan, 2005) and is still widely used to improve the symptoms of PD patients. The reason behind this choice should be attributed to TCM theory. Traditionally, motor symptoms like tremor and rigidity are ascribed to the dysfunction of the “Liver system,” and *P. lactiflora* could “nourish and tranquillize the liver system” to alleviate the symptoms (You, 2009). Literature suggests that *P. lactiflora* has crucial influence on the nervous and immune systems, and the therapeutic effects can be described as neuroprotective, antidepressant, sedative, analgesic, and anticonvulsant (Tan et al., 2020). According to the *Pharmacopoeia of the People’s Republic of China (2010)*, indications of *P. lactiflora* involve excessive sweating, abdominal pain, contracture of limbs, headache and dizziness, irregular menstruation, et al. (National Pharmacopoeia Commission, 2010). Diarrhea was reported as the most common adverse effect of *P. lactiflora* extracts (Feng et al., 2019); hence it is sometimes used for constipation. Modern studies have found that the Total Glucosides of Paeony (TGP), a capsule form extracted from *P. lactiflora,* exerts neuroprotective effect in MPTP-induced PD mice by activating cAMP/PKA/CREB on apoptosis pathway (Zheng et al., 2019), and paeoniflorin can reduce the neurotoxicity induced by glutamate in pheochromocytoma (PC12) cells (Sun et al., 2012). Furthermore, TGP capsule has a safe history of practice in the disease-modifying treatment of rheumatoid arthritis since being approved on market in China in 1998 (Luo et al., 2017), with anti-inflammatory and immunomodulatory effects (Zhang and Wei, 2020). Therefore, *P. lactiflora* may be a highly promising drug for PD.

Network pharmacology is a novel approach to analyze drug mechanisms, identify new targets, and expand new indications at a systematic level, which can comprehensively reflect the mechanism of drugs on disease networks, thus providing support for drug discovery for complex diseases and natural products (Hopkins, 2007; Kibble et al., 2015). Network pharmacology has become an effective method to predict the mechanism of TCM herbs and decoctions in the treatment of neurodegenerative diseases such as PD (Ke et al., 2016; Li et al., 2020). Therefore, this study intends to elucidate the underlying mechanism of *P. lactiflora* in the treatment of PD based on a network pharmacology approach from a holistic view.

## Materials and Methods

### Ingredients and Targets Screening of *Paeonia lactiflora*

To obtain the active ingredients of *P. lactiflora*, an initial screening based on the absorption, distribution, metabolism, and excretion (ADME) properties [oral availability (OB)≥ 30% and drug-likeness (DL) ≥ 0.18], was conducted by the TCM Systems Pharmacology (TCMSP, http://tcmspw.com/tcmsp.php) database (Ru et al., 2014). OB represents the proportion of oral medication absorbed into the circulation, and DL is a filter to exclude the non-drug-like molecules. These early-stage pharmacokinetic evaluations are of vital importance to reduce the probability of unsuccessful drug discovery (Wang and Urban, 2004). Since pathological and animal evidence have supported that PD may originate from the gut and spread to the central nervous system (Klingelhoefer and Reichmann, 2015), it is speculated that drugs may function outside the brain, so the blood-brain barrier permeability was not used as a criterion for drug screening. The names and structure formulas of compounds were verified using PubChem (https://pubchem.ncbi.nlm.nih.gov/) and the concentration of active components in this study were retrieved from literature.

Next, the known targets of these ingredients were collected from TCMSP and Swiss Target Prediction (http://www.swisstargetprediction.ch/) database (Gfeller et al., 2014). After screening, protein targets were standardized as gene symbol in Uniprot database (UniProt Consortium, 2019).

### Disease Targets Acquisition of Parkinson’s Disease

Using “PD” as the search term, we merged the obtained disease targets from three databases, OMIM (http://www.omim.org) (Amberger et al., 2015), Disgenet (https://www.disgenet.org/) (Piñero et al., 2017), and Drugbank (https://www.drugbank.ca) (Wishart et al., 2018), followed by deduplication.

### Protein-Protein-Interaction Network Analysis

We acquired the common targets of the drug and disease by intersection and then constructed a Protein-Protein-Interaction (PPI) network on the STRING11.0 database (https://string-db.org) (Szklarczyk et al., 2017). Set the biological species as *“Homo sapiens”* and hide the unconnected nodes. In addition, the network topology parameters were visualized and analyzed by CytoScape3.7.2 (Shannon et al., 2003) to identify the core targets. The Degree value represents the number of nodes connected by a node, so the higher the Degree of a node, the more important it is in the network. If there were too many targets, a PPI core network would be extracted twice with nodes whose Degree, Betweenness centrality (BC), and Closeness centrality (CC) values were all greater than the median.

### Gene Ontology Biological Process and Kyoto Encyclopedia of Genes and Genomes Enrichment Analysis

To illustrate the targets on a systematic level, the Gene Ontology Biological Process (GOBP) and Kyoto Encyclopedia of Genes and Genomes (KEGG) pathway enrichment analysis were carried out on the Metascape platform (http://metascape.org/gp/index.html). Metascape platform has integrated over 40 knowledgebases with monthly data updates and friendly interactive interface (Zhou et al., 2019). Items with a *p*-value < 0.01, count ≥ 3, and enrichment factor > 1.5 were collected and grouped into clusters based on their similarities automatically by the platform.

### Ingredient-Target-Pathway Network Analysis

To investigate the interaction among the active ingredients, common targets and the key KEGG pathway, the Ingredient-Target-Pathway (I-T-P) network of *P. lactiflora* for PD was constructed and visualized by CytoScape3.7.2. The main active components were judged according to the network topology parameters which was analyzed by its built-in network analyzer.

## Results

### Ingredients and Targets Screening of *Paeonia lactiflora*

11 active ingredients were extracted from 85 ingredients of *P. lactiflora* by ADME screening (Table 1). After deleting the duplicate targets obtained by TCMSP database and Swiss Target Prediction, a total of 240 targets of *P. lactiflora* were collected.

**TABLE 1:** Chemical properties of *Paeonia lactiflora* Pall. active ingredients.

Code	Molecular name	InCHI key	MOLID	Molecular formula	Structure formula	MW(g/mol)	OB (%)	DL	Concentration (mg/g)
PLP1	paeoniflorin	YKRGDOXKVOZESV-WRJNSLSBSA-N	MOL001924	C_23_H_28_O_11_		480.5	53.87	0.79	26.27 (Liu et al., 2015)
PLP2	Palbinone	KIAKLFLISZCITK-PPAUHQMUSA-N	MOL001919	C_22_H_30_O_4_		358.5	43.56	0.53	1.66 (Kadota et al., 1993)
PLP3	kaempferol	IYRMWMYZSQPJKC-UHFFFAOYSA-N	MOL000422	C_15_H_10_O_6_		286.24	41.88	0.24	Identified (Shu et al., 2014)
PLP4	(+)-catechin	PFTAWBLQPZVEMU-DZGCQCFKSA-N	MOL000492	C_15_H_14_O_6_		290.27	54.83	0.24	0.37 (Liu et al., 2015)
PLP5	betulinic acid (mairin)	QGJZLNKBHJESQX-FZFNOLFKSA-N	MOL000211	C_30_H_48_O_3_		456.78	55.38	0.78	N/A
PLP6	beta-sitosterol	KZJWDPNRJALLNS-VJSFXXLFSA-N	MOL000358, MOL00359	C_29_H_50_O		414.7	36.91	0.75	Identifie d (Wu et al., 2020)
PLP7	paeoniflorgenone	BANPEMKDTXIFRE-GHVWTTSJSA-N	MOL001918	C_17_H_18_O_6_		318.35	87.59	0.37	identified (Wu et al., 2020)
PLP8	albiflorin_qt	WSVOZDIRZKFUCH-UNTBTUBKSA-N	MOL001928	C_23_H_28_O_11_		318.35	66.64	0.33	9.83 (Liu et al., 2015)
PLP9	benzoyl paeoniflorin	KHRHASRIMPQOPU-JEWJNOKWSA-N	MOL001930	C_30_H_32_O_12_		584.62	31.27	0.75	0.39 (Liu et al., 2015)
PLP10	11alpha,12alpha-epoxy-3beta-23-dihydroxy-30-norolean-20-en-28,12beta-olide	PEQYYKKHHKGVKL-NZIMWEAXSA-N	MOL001910	N/A		470.71	64.77	0.38	N/A
PLP11	lactiflorin	KEMSOUIGHYEWRY-UAJYNZJHSA-N	MOL001921	C_23_H_26_O_10_		462.49	49.12	0.8	Identified (Yu et al., 1990)

Notes: PLP, *Paeonia lactiflora* Pall.; MW, Molecular Weight; OB, Oral Availability; DL: Drug-likeness.

### Disease Targets Acquisition of Parkinson’s Disease

523 targets from OMIM, 97 from Drugbank, and 1,063 from Disgenet database were integrated and finally 1607 PD targets were obtained.

### Protein-Protein-Interaction Network Analysis

**FIGURE 1:**
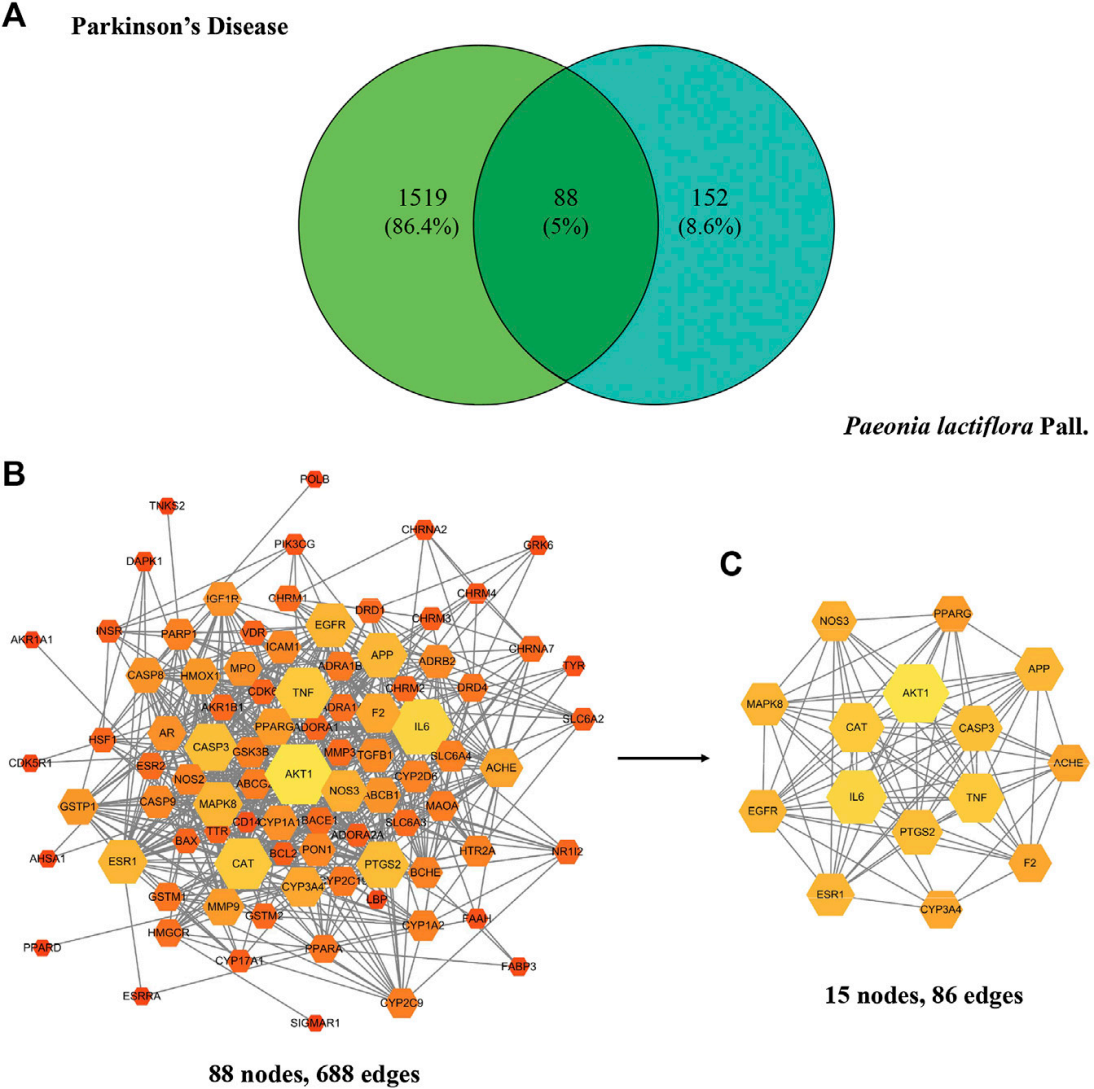
**(A)** Common targets of *Paeonia lactiflora* Pall. and Parkinson’s disease. **(B)** Protein-Protein-Interaction (PPI) network of putative targets. **(C)** PPI core network. The size and color of the node represent its Degree. Degree equals the number of nodes connected by a node. The larger and brighter the node is, the more important it is in the network.

A Venn diagram was rendered to show the 88 common targets between *P. lactiflora* ingredients and PD (Figure 1A). In the String database, PPI network was established consisting of 88 nodes and 688 edges (Figure 1B). Based on the analysis of the topological characteristics of PPI network, AKT1 was the most important target in the network (Degree = 51, BC = 0.1374, CC=0.7073). To make it clear, PPI core network of 15 nodes and 86 edges was further extracted (Figure 1C). Consequently, top 6 targets, AKT1, interleukin-6 (IL-6), CAT, Tumor necrosis factor (TNF), CASP3, and PTGS2, were predicted to be the key targets of *P. lactiflora* in the treatment of PD.

### Gene Ontology Biological Process and Kyoto Encyclopedia of Genes and Genomes Enrichment Analysis

**FIGURE 2:**
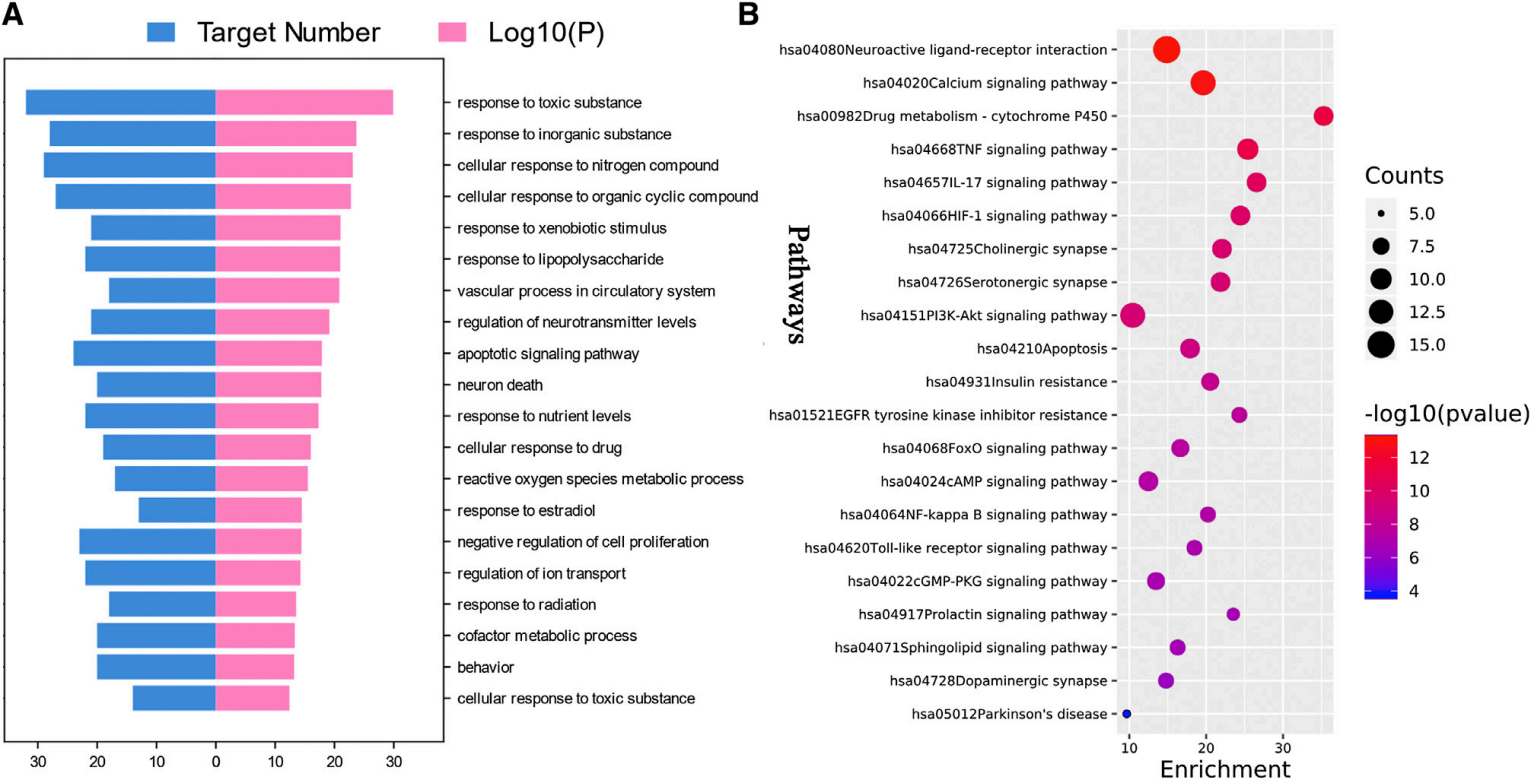
Enrichment analysis of putative targets of *Paeonia lactiflora* P. for Parkinson’s disease. **(A)** Top 20 Gene Ontology biological processes. **(B)** Top 20 Kyoto Encyclopedia of Genes and Genomes (KEGG) pathways and Parkinson’s disease Pathway. Size of the bubble stands for the number of targets in the pathway and the color stands for *p* value. The red bubble represents a smaller *p* value. (*p* < 0.01).

Through the enrichment analysis of 88 common targets on the Metascape platform, 1,436 GOBP items were collected and clustered by the platform, and top 20 clusters were selected and arranged according to the ascending order of *p* value, as shown in Figure 2A. Results show that the main biological processes involved the response to toxic substances, regulation of neurotransmitter levels, apoptosis signaling pathway, neuron death, reactive oxygen species (ROS) metabolic process, and so on.

After removing pathways of other diseases, the top 20 KEGG pathways with the lowest p value and PD signaling pathway were shown in Figure 2B. The main pathways involved were neuroactive ligand-receptor interaction, Calcium signaling pathway, P450 Drug metabolism-cytochrome P450, TNF signaling pathway, IL-17 signaling pathway, HIF-1 signaling pathway, cholinergic synapse, serotonergic synapse, PI3K-Akt signaling pathway, and apoptosis. Details were listed in Table 2. Moreover, the PD signaling pathway was also observed, with five targets namely ADORA2A, CASP3, CASP9, DRD1, and SLC6A3.

**TABLE 2:** Kyoto Encyclopedia of Genes and Genomes pathway enrichment of *Paeonia lactiflora* P. for PD.

Gene ontology	Description	Log_P_	Counts	Hits
hsa04080	Neuroactive ligand-receptor interaction	−13.10	15	ADORA1, ADORA2A, ADRA1B, ADRA1A, ADRB2, CHRM1, CHRM2, CHRM3, CHRM4, CHRNA2, CHRNA7, DRD1, DRD4, F2, HTR2A
hsa04020	Calcium signaling pathway	−12.92	13	ADORA2A, ADRA1B, ADRA1A, ADRB2, CHRM1, CHRM2, CHRM3, CHRNA7, DRD1, EGFR, HTR2A, NOS2, NOS3
hsa00982	Drugmetabolism-cytochromeP450	−11.39	9	CYP1A2, CYP2C19, CYP2C9, CYP2D6, CYP3A4, GSTM1, GSTM2, GSTP1, MAOA
hsa04668	TNF signaling pathway	−11.15	10	AKT1, CASP3, CASP8, ICAM1, IL-6, MMP3, MMP9, MAPK8, PTGS2, TNF
hsa04657	IL-17 signaling pathway	−10.25	9	CASP3, CASP8, GSK3B, IL-6, MMP3, MMP9, MAPK8, PTGS2, TNF
hsa04066	HIF-1 signaling pathway	−9.93	9	AKT1, BCL2, EGFR, HMOX1, IGF1R, IL-6, INSR, NOS2, NOS3
hsa04725	Cholinergic synapse	−9.52	9	ACHE, AKT1, BCL2, CHRM1, CHRM2, CHRM3, CHRM4, CHRNA7, PIK3CG
hsa04726	Serotonergic synapse	−9.49	9	APP, CASP3, CYP2C19, CYP2C9, CYP2D6, HTR2A, MAOA, PTGS2, SLC6A4
hsa04151	PI3K-Akt signaling pathway	−9.47	13	AKT1, BCL2, CASP9, CDK6, CHRM1, CHRM2, EGFR, GSK3B, IGF1R, IL-6, INSR, NOS3, PIK3CG
hsa04210	Apoptosis	−8.71	9	PARP1, AKT1, BAX, BCL2, CASP3, CASP8, CASP9, MAPK8, TNF
hsa04931	Insulin resistance	−8.26	8	AKT1, GSK3B, IL-6, INSR, NOS3, PPARA, MAPK8, TNF
hsa01521	EGFR tyrosine kinase inhibitor resistance	−7.81	7	AKT1, BAX, BCL2, EGFR, GSK3B, IGF1R, IL-6
hsa04068	FoxO signaling pathway	−7.54	8	AKT1, CAT, EGFR, IGF1R, IL-6, INSR, MAPK8, TGFB1
hsa04024	cAMP signaling pathway	−7.34	9	ADORA1, ADORA2A, ADRB2, AKT1, CHRM1, CHRM2, DRD1, PPARA, MAPK8
hsa04064	NF-kappaB signaling pathway	−7.24	7	PARP1, BCL2, CD14, ICAM1, LBP, PTGS2, TNF
hsa04620	Toll-like receptor signaling pathway	−6.97	7	AKT1, CASP8, CD14, IL-6, LBP, MAPK8, TNF
hsa04022	cGMP-PKG signaling pathway	−6.83	8	ADORA1, ADRA1B, ADRA1A, ADRB2, AKT1, INSR, NOS3, PIK3CG
hsa04917	Prolactin signaling pathway	−6.67	6	AKT1, CYP17A1, ESR1, ESR2, GSK3B, MAPK8
hsa04071	Sphingolipid signaling pathway	−6.60	7	ADORA1, AKT1, BAX, BCL2, NOS3, MAPK8, TNF
hsa04728	Dopaminergic synapse	−6.31	7	AKT1, DRD1, DRD4, GSK3B, MAOA, MAPK8, SLC6A3
hsa05012	Parkinson's disease	−3.76	5	ADORA2A, CASP3, CASP9, DRD1, SLC6A3

IL-6, Interleukin 6; TNF, Tumor Necrosis Factor; IL-6, Interleukin 6; CASP8, Caspase-8; CASP9, Caspase-9; CASP3, Caspase-3; PTGS2, Cyclooxygenase 2 (COX2); BCL2, Apoptosis regulator Bcl-2; AKT1, RAC-alpha serine/threonine-protein kinase (PKB); ADORA2A, Adenosine receptor.

### Ingredient-Target-Pathway Network Analysis

**FIGURE 3:**
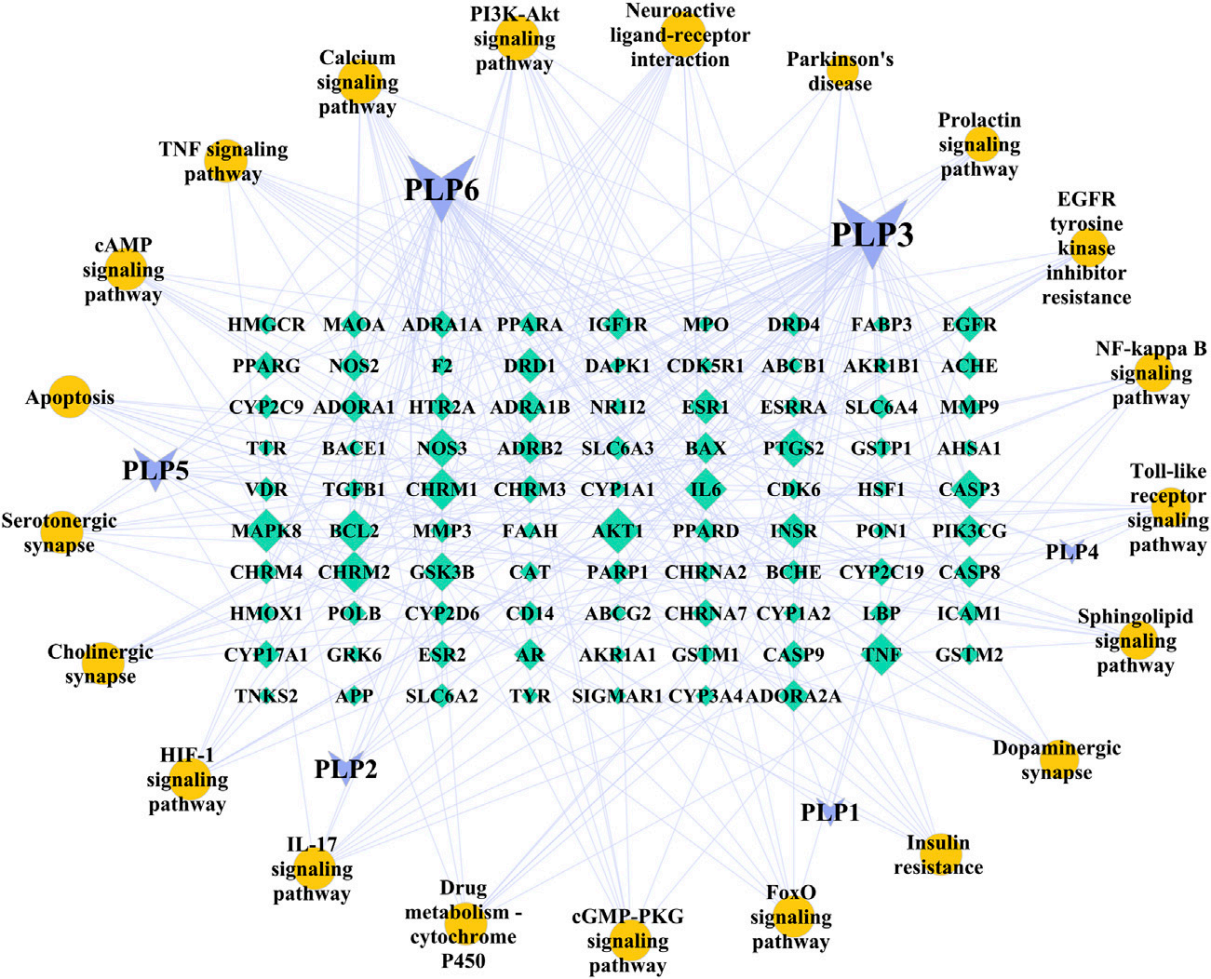
Ingredient-Target-Pathway (I-T-P) network of *Paeonia lactiflora* P. for Parkinson’s disease. The purple inverted triangles represent the ingredients of *Paeonia lactiflora* P., the green diamonds represent the common targets, and the yellow circles represent the main pathways. Node size is proportional to its degree. The larger the area is, the more important it is in the network. The nodes of ingredients and pathways are arranged clockwise by size. PLP1, paeoniflorin; PLP2, palbinone; PLP3, kaempferol; PLP4, (+)-catechin; PLP5, betulinic acid; PLP6, beta-sitosterol.

As shown in Figure 3, six core ingredients were obtained, which were kaempferol, ß-sitosterol, betulinic acid, palbinone, paeoniflorin, and (+)-catechin in turn. Kaempferol was the most important ingredient (Degree = 57, BC = 0.5483, and CC = 0.5643), followed by ß-sitosterol (52, 0.2646, 0.4634). In addition, AKT1, mitogen-activated protein kinase 8(MAPK8), BCL2, TNF, and IL-6 were found to be the top five important targets in this network.

## Discussion

**FIGURE 4:**
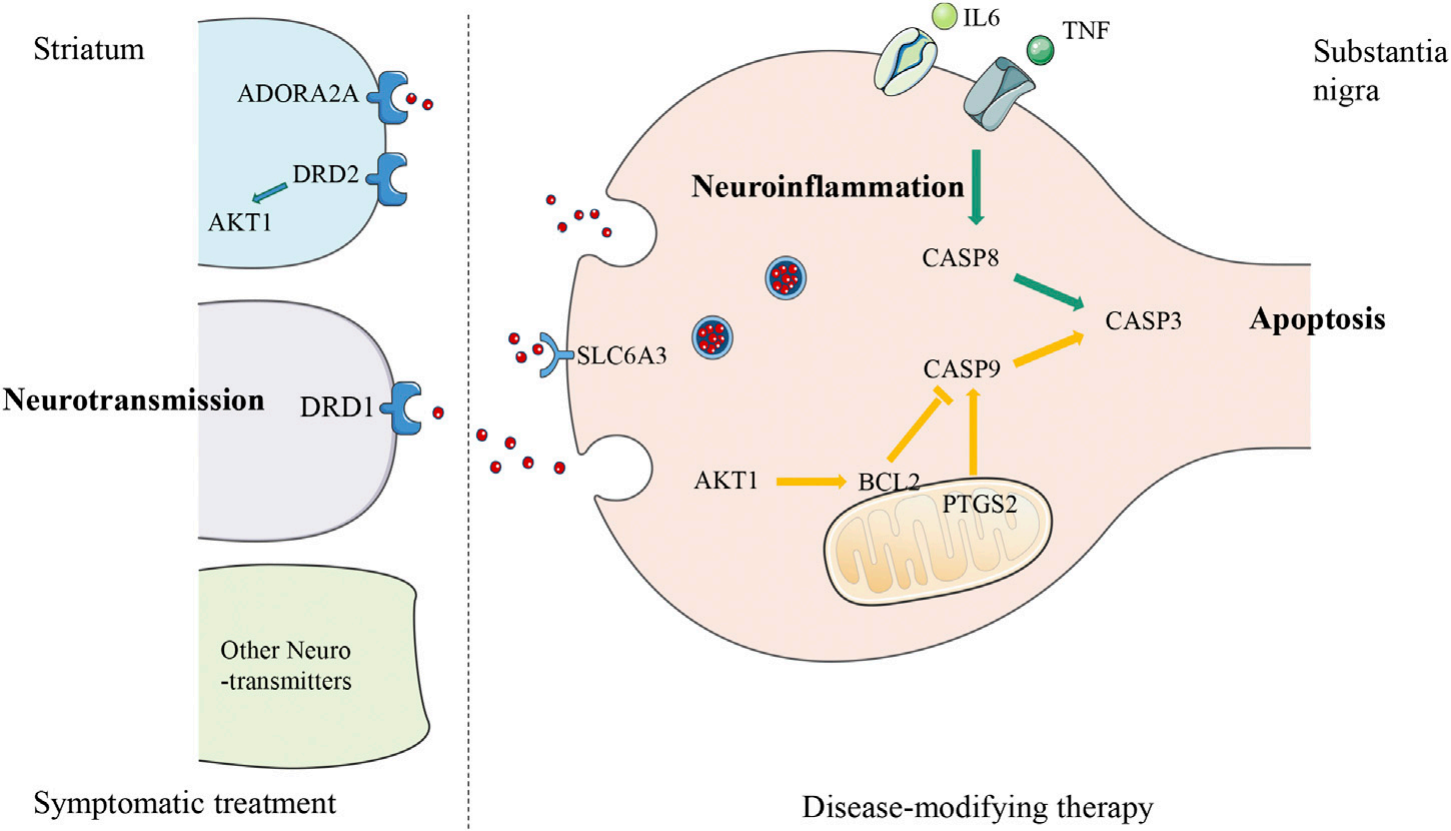
Schematic diagram of the underlying mechanism of *Paeonia lactiflora* P. in Parkinson’s disease. The diagram of a nigro-striatal synapse shows three possible approaches that can be used as therapeutic targets. Parkinson’s disease is caused by the early death of dopaminergic neurons in midbrain substantia nigra leading to the reduction of dopamine neurotransmitters released to the striatum, so treatment could be either targeting the degeneration process as disease-modifying therapy (presynaptic, right side of the diagram), or synaptic transmission as symptomatic treatment (postsynaptic, left side of the diagram). The putative targets of *Paeonia lactiflora* P. are located on three pathways: the green arrows from TNF, IL-6, to CASP8, CASP3 are part of the extrinsic apoptosis pathway induced by neuroinflammation, the yellow arrows from CASP9 to CASP3 with the involvement of AKT1, BCL2, and PTGS2, belong to the intrinsic apoptosis pathway, and blue receptors DRD1, DRD2, ADORA2A, SLC6A3 and AKT1 are related to the regulation of neurotransmitters level. TNF, Tumor Necrosis Factor; IL-6, Interleukin 6; CASP8, Caspase-8; CASP9, Caspase-9; CASP3, Caspase-3; PTGS2, cyclooxygenase 2 (COX2); BCL2, Apoptosis regulator Bcl-2; AKT1, RAC-alpha serine/threonine-protein kinase (PKB); ADORA2A, Adenosine receptor A2a; DRD1, Dopamine D1 receptor; DRD2, Dopamine D2 receptor; SLC6A3, Sodium-dependent dopamine transporter.

Despite substantial advances in the molecular mechanism of PD, conventional clinical trials of single gene and single channel drugs have failed to slow the progression of PD (Olanow et al., 2009; Schapira et al., 2013; Verschuur et al., 2019). The possible reasons are as follows: first, the etiology of PD, involving complex genetic and environmental factors, is difficult to identify, very likely due to highly individualized reasons (Ramsay et al., 2016). Secondly, PD pathology is widely distributed in both central nervous system and peripheral nervous system (Braak et al., 2004), affecting several neurotransmitter systems other than dopaminergic (Sanjari Moghaddam et al., 2017). Therefore, one of the latest trends in the research of disease-modifying drugs is the reasonable combination of multiple targets (Kalia and Lang, 2015; Ke et al., 2016). Coincidentally, TCM exerts its effect through multiple targets and pathways with multiple components, which provides symptomatic relief for PD patients and shows neuroprotective effect on rodents *in vivo* and *in vitro*. Extensive use of *P. lactiflora* in TCM clinics aroused our interest in this herb, and then a network pharmacology approach was adopted to explore its mechanism on a holistic view. In this study, seven active ingredients, six core targets of *P. lactiflora* and related pathways in the treatment of PD were identified. A Schematic diagram of the underlying mechanism is shown in Figure 4.

### *Paeonia lactiflora* may Contribute to the Treatment of Parkinson’s Disease by Reducing Neuroinflammation

Abundant evidence supports that neuroinflammation is an important factor of PD whereby oxidative stress and apoptosis caused by neuroinflammatory response can damage dopaminergic neurons (Hirsch and Hunot, 2009; Guzman-Martinez et al., 2019). Epidemiological investigations also found that non-steroidal anti-inflammatory drugs (NSAIDs) are related to a lower risk of developing PD (Noyce et al., 2012), suggesting that neuroinflammation may promote disease progression. In addition, emerging evidence demonstrates that intestinal inflammation may also be involved in PD pathophysiology through the regulation of the gut-brain axis (Chen et al., 2019).

We found that the TNF signaling pathway is a key pathway of *P. lactiflora* for PD. Initiated from a potent pro-inflammatory cytokine TNF, TNF signaling pathway mediates a wide range of cellular processes including inflammation, proliferation, cell migration, apoptosis, and necrosis (McCoy and Tansey, 2008). After TNF binds to TNF Receptor 1 (TNFR1), the adaptor proteins including receptor interacting protein and TNF receptor associated factor 2 (TRAF2) will form a complex (Hsu et al., 1996). This complex can trigger the activation of a series of signaling pathways downstream, including nuclear factor kappa-B (NF-κB) signaling pathway, MAPK signaling pathway, and ubiquitin proteasome signaling pathway (Winston et al., 1995). Translocation of NF-κB to the nucleus is a crucial step where cell survival genes, pro-inflammatory cytokines, chemokines, growth factors and TNFα itself were transcribed. Many of these activities participate in inflammation, such as IL-6 production, cyclooxygenase-2 (COX2) synthesis, extracellular matrix remodeling, leukocyte recruitment, (Newton and Dixit, 2012). In addition, autopsy study of PD patients found up-regulation of TNFR1 expression on dopaminergic neurons, and activation of central glial cells and peripheral immune cells (Mogi et al., 1996). A recent research showed that the effect of an NF-κB inhibitor (50 mg/kg) is comparable to levodopa-carbidopa combination in lipopolysaccharide (LPS) induced rats, with a decrease in TNF-α and IL-6 levels (Saini et al., 2020). These findings further confirm that inhibition of TNF signaling pathway might be associated with reducing neuroinflammation in PD. According to the results of I-T-P network analysis, kaempferol and paeoniflorin in *P. lactiflora* may reduce neuroinflammation by inhibiting the expression of TNF and IL-6 in the treatment of PD.

### *Paeonia* *lactiflora* may Contribute to the Treatment of Parkinson’s Disease by Inhibiting Apoptosis

Apoptosis is proved to be a death mode of neurons in substantia nigra in PD patients. Unlike cell necrosis, apoptosis is an active death process mediated by intrinsic or extrinsic signaling pathways, regulated by caspase family, apoptosis regulator (BCL2), family and other genes (Perier et al., 2012).

We found that AKT1, CASP3 and PTGS2 are the key targets of *P. lactiflora* in the treatment of PD, and BCL2 is also among the targets. AKT, also known as protein kinase B, plays a key role in a variety of signaling pathways with more than 100 substrates. AKT phosphorylation induces the binding of accessory proteins necessary for the anti-apoptosis gene BCL2 expression, which in turn inhibits apoptosis by regulating the permeability of the mitochondrial outer membrane (Hers et al., 2011). Another target PTGS2, also known as COX2, is a key prostaglandin synthase induced by inflammation, which is also the target of NSAIDs such as ibuprofen. Generally, PTGS2 is not expressed in dopaminergic neurons, while it is significantly positive in PD patients and mouse models (Teismann et al., 2003). Evidence proves that COX2 inhibition mitigates BCL2, Caspase-9 (CASP9), and Caspase-3 (CASP3) expression through the intrinsic apoptosis pathway (Chauhan et al., 2018). In addition, in the aforementioned inflammatory signaling pathway, TNF/TNFR1 can also bind to FAS-associated death domain protein , activating extrinsic apoptosis pathway, and further leads to apoptosis through the activation of key proteolytic enzymes Caspase-8 (CASP8) and CASP3 (McCoy and Tansey, 2008). According to the results of I-T-P network analysis, kaempferol in *P. lactiflora* may regulate AKT1, CASP3, and BCL2, as well as kaempferol, ß sitosterol, and (+)-catechin may regulate PTGS2 to inhibit intrinsic and extrinsic apoptosis in the treatment of PD.

### *Paeonia lactiflora* may Improve the Motor Symptoms and Non-Motor Symptoms of Parkinson’s Disease by Regulating Multiple Neurotransmitters level

According to the classical model of basal ganglion circuit, dyskinesia in PD is caused by unbalanced activity of direct and indirect pathways. The decline of dopamine transmitter released from the substantia nigra to the striatum leads to a decrease in the excitability of the direct pathway (dopamine D1 receptor, DRD1) and the over-activation of the indirect pathway (dopamine D2 receptor, DRD2; adenosine receptor A2a, ADORA2A), which collectively suppress the cortical excitability (McGregor and Nelson, 2019). A2A receptor antagonists is a non-dopamine drug in advanced PD which can shorten the “off” time and improve motor symptoms (Mizuno et al., 2013). AKT1, located downstream of the DRD2, is also involved in the dopamine signaling cascade and affect the expression of dopamine-related behavior in the striatum (Beaulieu et al., 2007). The Sodium-dependent dopamine transporter (SLC6A3) is in charge of dopamine concentration regulation by mediating reuptake on the presynaptic membrane (Habak et al., 2014). I-T-P network analysis showed that *P. lactiflora* may regulate DRD1, ADORA2A, and SLC6A3 in the PD pathway and AKT1 downstream to relieve motor symptoms.

NMS are key determinants of quality of life. However, limited treatments are available (Schapira et al., 2017). Mainstream dopaminergic therapy has no effect on NMS caused by defects in other neurotransmitter pathways, which even aggravates the problem such as constipation (Schaeffer and Berg, 2017). Clinical use of selective serotonin receptor (5-HT) agonists, such as Mosapride, suggests damage to serotonergic nerves (Sakakibara et al., 1996). Selective anticholinergic drugs such as Solifenacin may have an effect on overactive bladder syndrome (Zesiewicz et al., 2015). The enrichment analysis indicates that neuroactive ligand-receptor interaction is the most important pathway of *P. lactiflora* for PD, as well as serotonergic synaptic pathway, cholinergic synaptic pathway, and dopaminergic synaptic pathway also being observed, suggesting that *P. lactiflora* may relieve NMS through the regulation of a variety of neurotransmitter pathways.

Likewise, several studies have clearly demonstrated the neuroprotective effects of *P. lactiflora* components. For example, kaempferol inhibits the activation of NLRP3 inflammasome through the synergistic effect of ubiquitin and autophagy, thus promotes the survival of dopaminergic neurons in PD models induced by LPS and SNCA (Han et al., 2019). Kaempferol can also maintain the stability of blood-brain barrier and down-regulate HMGB1/TLR4 pathway to reduce striatal injury in mice (Yang et al., 2019). A number of studies showed that paeoniflorin protects PC12 cells against MPP+, acid, and glutamate induced neuron injury via regulating autophagy, apoptosis, and mitochondrial membrane potential (Cao et al., 2010; Sun et al., 2012; Zheng et al., 2016). It also reduces dopaminergic neurodegeneration in MPTP model by inhibition of neuroinflammation and suppression of the accelerated dopamine catabolism via Amine oxidase B (MAOB) inhibition (Liu et al., 2006; Zheng et al., 2017). ß-sitosterol enhances membrane potential and ATP content of mitochondria which is expected to benefit AD (Shi et al., 2013). Betulinic acid, also known as mairin, induces apoptosis in cells through the mitochondrial pathway mediated by ROS (Wang et al., 2017).

Besides anti-parkinsonian property, *P. lactiflora* also exhibits neuroprotective potential in other neurodegenerative diseases. Acetylcholinesterase (AChE) inhibitor is a first-line treatment for AD dementia, and possibly useful for PD dementia (Seppi et al., 2019). Lin compared the AChE inhibitory activities among 26 herbs *in vitro*, while ethanol extracts of *P. lactiflora* roots showed the second strongest inhibition (IC_50_ = 8 μg/ml) in a dose-dependent manner (Lin et al., 2008). Albiflorin, one of the core components of *P. lactiflora*, ameliorates memory deficits and reduces brain amyloid-β deposition in APP/PS1 mice (Xu et al., 2019). Above mentioned TGP can reduce the severity and progression of experimental autoimmune encephalomyelitis, a multiple sclerosis model in mice, by attenuating inflammation response (Huang et al., 2015).

Among more than 50 types of plants against MPTP-induced neurotoxicity in a recent review, antioxidant, antiapoptotic, and autophagy enhancement are the most studied mechanisms (Abushouk et al., 2017). *P. lactiflora* and Mucuna pruriens seeds have similar mechanism in ameliorating neuroinflammation and apoptosis, while the latter contains natural levodopa used as a dopamine supplement in India (Lieu et al., 2012; Rai et al., 2017). Both *P. lactiflora* and Sophora Tomentosa exert antioxidant properties, which might be associated with their common component, catechin (Chang et al., 2019). Although *P. lactiflora* is a major component in several formulas reported to be neuroprotective *in vivo* (Ahn et al., 2019; Tang et al., 2020), this is the first time to explore its mechanism alone from a systematic level, which may benefit further drug manufacturing.

Our study has several limitations. First, since the network pharmacology approach is performed by target prediction based on existing studies, the unique ingredients of *P. lactiflora* like paeoniflorin is less recognized compared to common ingredients like kaempferol, which may affect the importance of the components in the target network. Secondly, further experiments should verify the conclusion drawn in this study, including the pathways, neurotransmitters level, and neuron degeneration. Another interesting question to answer is whether the components play a role through the peripheral or central nervous system. Future research aimed toward novel drug development must consider safety as well as efficacy of the components; and down the line, do the results obtained in cellular or animal models translate to the bedside?

## Conclusion

Taken together, *P. lactiflora* may retard neurodegeneration by reducing neuroinflammation, inhibiting intrinsic and extrinsic apoptosis, and improve motor and NMS by regulating the level of neurotransmitters. Our study has revealed the underlying multicomponent, multitarget, and multipathway mechanism of *P. lactiflora* in the treatment of PD, which may contribute to novel drug development for PD.

## Data Availability Statement

The raw data supporting the conclusions of this article will be made available by the authors, without undue reservation.

## Author Contributions

WD, YZ conceived this work and drafted the manuscript, XL, SW collected the data, and PL assisted in the revision of the manuscript. All the authors revised and approved the manuscript.

## Funding

This research was supported by the National TCM Leading Personnel Support Program (NATCM Personnel and Education Department [2018] No.12).

## Conflict of Interest

The authors declare that the research was conducted in the absence of any commercial or financial relationships that could be construed as a potential conflict of interest.
